# Distinct structural interactions of polyadenine and polythymine on gold nanoparticles: from single strands to duplexes

**DOI:** 10.1039/d5sc04459f

**Published:** 2025-07-31

**Authors:** Manuel Núñez-Martínez, Jinyi Dong, Isabel García, Bjorn De Busschere, Nathalie Claes, Sara Bals, Luis M. Liz-Marzán

**Affiliations:** a CIC biomaGUNE, Basque Research and Technology Alliance (BRTA) 20014 Donostia-San Sebastián Spain mnunez@cicbiomagune.es igarcia@cicbiomagune.es llizmarzan@cicbiomagune.es; b Networking Biomedical Research Center, Bioengineering, Biomaterials and Nanomedicine (CIBER-BBN) 20014 Donostia-San Sebastián Spain; c Ikerbasque 48009 Bilbao Spain; d EMAT and NANOlab Center of Excellence, University of Antwerp B-2020 Antwerp Belgium sara.bals@uantwerpen.be; e CINBIO, University of Vigo 36310 Vigo Spain; f CAS Key Laboratory of Nano-Bio Interface, Suzhou Key Laboratory of Functional Molecular Imaging Technology, Division of Nanobiomedicine and i-Lab, Suzhou Institute of Nano-Tech and Nano-Bionics, Chinese Academy of Sciences Suzhou 215123 China

## Abstract

Motivated by potential applications in fields such as medicine or materials science, various methodologies have been developed for the preparation of so-called spherical nucleic acids, based on oligonucleotides and metal nanoparticles. Despite optimization through various parameters such as loading efficiency or nanoconjugate stability, much remains to be known regarding the actual conformations of oligonucleotides and their interactions with the nanoparticle surface. We employed a combination of spectroscopic techniques and liquid transmission electron microscopy to analyze the interactions and conformations adopted by polyAdenine (polyA) and polyThymine (polyT) chains in contact with gold nanoparticles (AuNPs). These studies revealed the presence of AuNP@polyA dimers, with polyA strands forming duplexes, whereas polyT forms isolated strands on the AuNPs. The presence or absence of polyA duplexes on AuNPs can be modulated by external stimuli such as temperature or NaCl. This study contributes to understanding the interactions and secondary structure of oligonucleotides on AuNPs.

## Abstract 2

The secondary structure of ssDNA on gold nanoparticles was determined by means of optical spectroscopic and advanced electron microscopy in a liquid environment.

## Graphical abstract

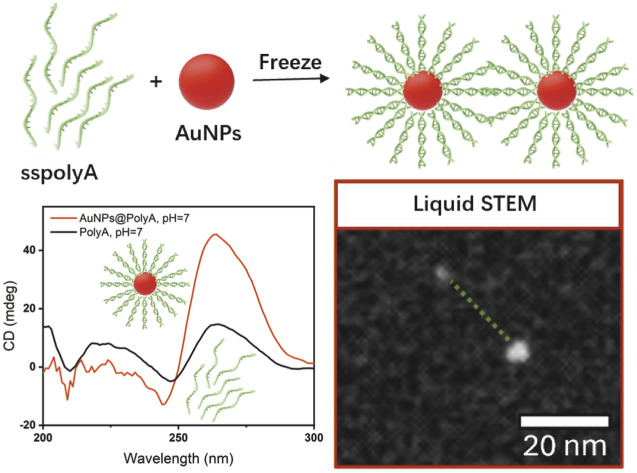

## Introduction

Spherical nucleic acids (SNAs) are nanostructures comprising a dense shell of nucleic acid strands, radially arranged around a nanoparticle core.^1,2^ Their unique properties offer potential advancements in medicine, diagnostics, and materials science.^3–6^ Since the development of multivalent gold nanoparticle-DNA (AuNP-DNA) conjugates in 1996, researchers have explored a wide range of metal cores with varying sizes, shapes, and compositions, along with shells differing in DNA chain length, density, and flexibility.^7–11^ SNAs additionally differ from traditional nucleic acids in their dense arrangement of oligonucleotides around an inorganic core, unlike the linear forms that they assume in hybridized duplexes.^12^

SNAs can be composed of both single-stranded nucleic acids and DNA frameworks, with their overall shape dictated by the metal core material.^13^ Different methodologies have been developed to prepare SNAs with different DNA densities, such as salt-aging, thermal drying, acoustic levitation, butanol, pH- or freeze-assisted protocols.^14–19^ On the other hand, a systematic exploration of the DNA secondary structures in SNAs, as well as understanding of inter-strand interactions on the nanoparticle surface, is still lacking because of the inherent complexity of the relevant surface characterization.

**Scheme 1:**
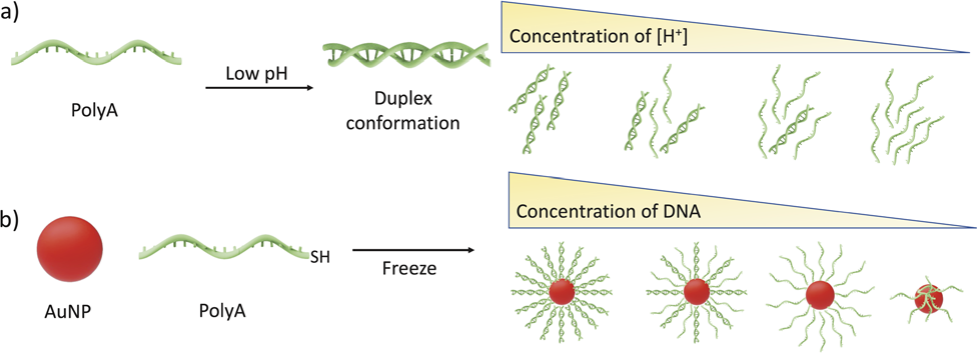
(a) Graphical illustration of secondary structures of polyA at different pH values. (b) Graphical illustration of AuNP@polyA, at different DNA concentrations on the gold surface.

Focusing on a particular case, single-stranded oligo-adenine (sspolyA) is known to form parallel duplex structures *via* protonated adenines, known as A-motifs,^20–22^ with pH-reversible protonation and deprotonation processes. sspolyA has been reported to show a dynamic behavior, so that its secondary structure can be modulated by external stimuli such as pH, salt addition, temperature, *etc.* For instance, when sspolyA is dissolved at low pH (*e.g.* pH = 3), it can form duplexes between two sspolyA. On the other hand, at higher pH (*e.g.* pH = 7) the duplexes are disrupted, leading to stabilization of sspolyA^21^ (Scheme 1a). A-motifs have demonstrated high efficiency in creating SNAs due to the decreased electrostatic repulsion between both negatively charged AuNPs and DNA strands (Scheme 1b). Previous studies showed that A-motif duplex-functionalized AuNPs at pH 3 can dissociate and form single polyA strands around the surface of AuNPs at pH 7.^23^ Furthermore, other studies used non-thiolated polyA for describing the mechanism of duplex formation by adsorption, as well as the role of polyA length in the stabilization of AuNPs.^8,22^ Herein, we found evidence of a stable A-motif made of high density polyA SNA on gold surfaces with high curvature (small particle size), dispersed in deionized water (di-H_2_O). Therefore, we pursued the origin of this structure, through re-evaluation of the freezing method and polyA-based affinity labeling. Characterization of the secondary structure and conformation was obtained by means of circular dichroism (CD), absorbance (UV-vis), and emission (photoluminescence) spectroscopies, as well as transmission electron microscopy (TEM) in liquid. Through the combination of *in situ* spectroscopic and electron microscopy techniques we observed the presence of AuNP@polyA dimers, with the polyA strands forming duplexes around the AuNP core. Interestingly, stable polyA duplexes are related to high DNA density on the AuNPs. A similar spectroscopy and liquid state electron microscopy approach was carried out using polyT as a control ssDNA with lower affinity for the Au surface. The obtained results confirmed a different behavior, where polyT formed isolated strands on AuNPs and no secondary structure was detected.

## Experimental

### Chemicals and materials

Sodium chloride, tetrachloroauric(iii) acid (≥99% trace metal basis), trisodium citrate dihydrate (≥99%), dl-dithiothreitol (DTT), citric acid monohydrate (≥99%), potassium carbonate (≥99%), and tannic acid (MW 1701, ACS reagent) were purchased from Sigma-Aldrich. All chemical reagents were used as received without further purification. DNA oligonucleotides were custom-synthesized by Sigma, and purified by desalting. The sequences of the oligonucleotides used in this work are provided in Table S1.

### Synthesis of AuNPs

150 mL of freshly prepared aqueous solutions of sodium citrate (2.2 mM) containing 0.1 mL of tannic acid (TA, 2.5 mM) and 1 mL of potassium carbonate (K_2_CO_3_, 150 mM) were heated with a heating mantle in a 250 mL three-necked round-bottom flask under vigorous stirring. When the temperature reached 70 °C, 1 mL of tetrachloroauric acid (HAuCl_4_, 25 mM) was injected and the solution color changed rapidly to black/gray (less than 10 s) and then to orange-red in the following 1–2 min. The solution was kept at 70 °C for an additional 5 min to ensure complete reduction of the gold precursor. The resulting particles (∼3.5 nm, 7 × 10^13^ NP per mL) were narrowly dispersed, negatively charged and stable for weeks.

### Conjugation of ssDNA to AuNPs

Freeze-thawing was carried out following protocols previously described in the literature.^14^ Briefly, thiolated ssDNA was added to citrate-capped AuNPs at a DNA : AuNP molar ratio of 20 : 1. The sample was subjected to freezing at −20 °C for 2 h. After removing the free DNA by ultrafiltration (5000 g, 2 min, five times), the amount of DNA on each AuNP was quantified based on the fluorescence method.

### Fluorometric quantification of grafted DNA on AuNPs

To quantify the number of grafted DNA strands, thiol/FAM dual-labeled DNA was used to prepare SNAs. The concentrations of AuNPs were spectroscopically measured based on the extinction coefficient of gold at 400 nm. Then, the obtained SNAs were incubated with DTT at 60 °C for 10 h to displace surface-grafted DNA. Then, AuNPs were removed by centrifugation to avoid their quenching effect, prior to fluorometric quantification of DNA density. Fluorescence was excited at 495 nm and emission was recorded between 500 and 650 nm. SNAs were prepared in three parallel batches to obtain an average DNA grafting density.

### Spectroscopy and microscopy characterization

Spectroscopy characterization was conducted with an Agilent 8453 UV-vis-NIR diode array spectrophotometer in the 400–1100 nm wavelength range. Circular dichroism (CD) measurements were performed using a Jasco J-1500 spectropolarimeter equipped with a 150 W Xe excitation lamp, air cooled, and a detector (EXPML-535) with a detection range of 400–1250 nm. The cuvettes used were Hellma ultra micro-105.200-QS, and the CD extinction of the particles was measured first, followed by standard extinction measurements. Fluorescence spectra were recorded on a Biotek Synergy H1 microplate reader.

Dried TEM images were obtained on a JEOL JEM-1400PLUS transmission electron microscope operating at 100 kV. For dried TEM imaging, 2 μL of purified sample was added onto glow-discharged carbon-coated TEM grids (Grids were glow-discharged for 3 minutes at 10 mA) and adsorbed for 3 minutes, and the remaining solution was removed with filter paper.

High Angle Annular Dark Field Scanning Transmission Electron Microscopy (HAADF-STEM) and Energy Dispersive X-ray spectroscopy (EDX) were performed on a Tecnai Osiris transmission electron microscope (Thermo Fisher Scientific) equipped with a super-X detector and operated at 200 kV. For imaging nanoparticles in a liquid environment, a silicon-based K-kit microchannel device was employed. This liquid cell featured a 100 nm channel gap and 30 nm thick silicon nitride windows, allowing for *in situ* visualization of nanoparticles. The nanoparticle suspension was introduced into the liquid cell *via* capillary action.

Time series electron microscopy images were used to study the motion of AuNP@polyA and AuNP@polyT suspended in a liquid environment. Each frame of the movie underwent post-processing to improve segmentation quality, including Gaussian blurring and Fourier-based filtering techniques. This enhanced the contrast and distinguishability of the particles throughout the varying contrast conditions of the time series due to the build-up of contamination, resulting in accurate particle location determination in each frame. The center position of each particle was then tracked across the time series. The resulting trajectories were compiled into scatter plots, which allowed us to analyze the motion of the particles under continuous electron beam exposure, enabling the study of beam-induced effects on the ligand structure surrounding the nanoparticles.

## Results and discussion

### Preparation and characterization of AuNP@polyA

**Fig. 1:**
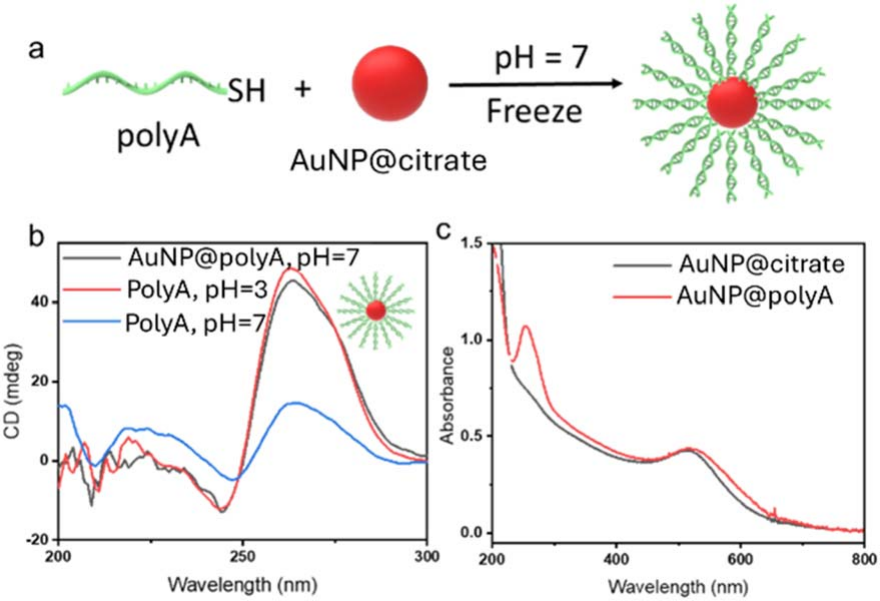
(a) Schematic illustration of the formation of AuNP@polyA duplexes on AuNPs. (b) CD spectra of AuNP@polyA at pH = 7 and free polyA strands at both pH = 3 and pH = 7. (c) UV-vis spectra of AuNP@citrate and AuNP@polyA.

We employed the freezing methodology to obtain AuNP@polyA conjugates (Fig. 1a). This method leads to the conjugation of thiolated ssDNA onto AuNPs after a freeze–thaw cycle, with high ssDNA density.^14^ Using this simple, fast and reagent-free strategy, a 20–30% higher DNA density can be obtained, as compared to other traditional methodologies such as salt-aging.^14^ A polyadenine segment comprising 45 nucleotides was selected because this polyA length allows interaction with the gold surface and/or the formation of secondary structures. Colloidally dispersed citrate-stabilized AuNPs (100 nM in AuNP, Abs_400_ = 0.39, 3.5 nm diameter, see TEM analysis in Fig. S1) were selected to reach a high density of surface ligands. The colloid was mixed with sspolyA at a sspolyA to AuNP ratio of 20 : 1 and this mixture was frozen at −20 °C for 2 hours. The mixtures were then thawed at room temperature and purified with di-H_2_O using 100k Amicon filters. Using this strategy, citrate molecules can be replaced by thiolated-sspolyA, obtaining the corresponding AuNP@polyA. After purification, the number of grafted sspolyA per AuNP was quantified, obtaining an average value of 12 strands per AuNP (Fig. S2).

Next, we proceeded to study the secondary structure of sspolyA in contact with the AuNP surface. We first noted that the CD spectrum of AuNP@polyA at pH = 7 shows an intense positive band around 260 nm (Fig. 1b), which is markedly different to the CD spectrum of the pure ssPolyA solution (1 μM in di-H_2_O) at the same pH, suggesting a change in polyA conformation upon adsorption onto small AuNPs. The concentration of AuNP@polyA was adjusted to 80 nM (as monitored by UV-vis spectroscopy) to obtain a concentration of PolyA on AuNPs close to that in the pure ssPolyA solution. Interestingly, an almost identical CD signal was recorded for AuNP@polyA (1 μM polyA in the dispersion) at pH = 7, compared with a polyA solution at pH = 3 (trisodium citrate buffer, 1 μM polyA). These results indicate that stable polyA duplexes are anchored by finite terminal polyA, leading to an increase of CD intensity around 260 nm. We thus conclude that the conjugation of polyA onto AuNPs affects significantly its secondary structure, resulting in the formation of polyA duplexes on the AuNP surface (Fig. 1a and b). As control experiments, non-thiolated polyA (45 bases) and other adenine-containing molecules were immobilized on gold surfaces using the same freezing process. Chiroptical characterization of non-thiolated polyA showed a weak CD band around 260 nm, compared to that for thiolated polyA, whereas the absorbance spectrum shows similar intensity at the plasmon resonance wavelength, confirming the adsorption of adenine on the gold surface without aggregation but not the presence of stable polyA duplexes around AuNPs (Fig. S3).^22^ The number of adenines in sspolyA is another key factor affecting the formation of this secondary structure, as exemplified in Fig. S4 for sspolyA with 11, 22, and 45 adenines.

The UV-vis spectrum of AuNP@polyA shows a well-defined band around 260 nm, again supporting the conjugation of sspolyA on the AuNP surface. We additionally observe a slight red-shift of the surface plasmon resonance band (compared to the initial AuNP@citrate colloid), from 509 to 515 nm, corresponding to an increase of the local refractive index upon DNA binding (Fig. 1c).^24^ We further performed Fourier transform infrared (FT-IR) spectroscopy measurements (Fig. S5), which showed a spectral red-shift for the peaks at 1661 and 1450 cm^−1^ (–NH_2_ and C=N functional groups respectively) in AuNP@polyA, related to inter- and intramolecular polyA interactions.^25,26^ The obtained results are therefore in agreement with our hypothesis of the formation of AuNP@polyA duplexes.

**Fig. 2:**
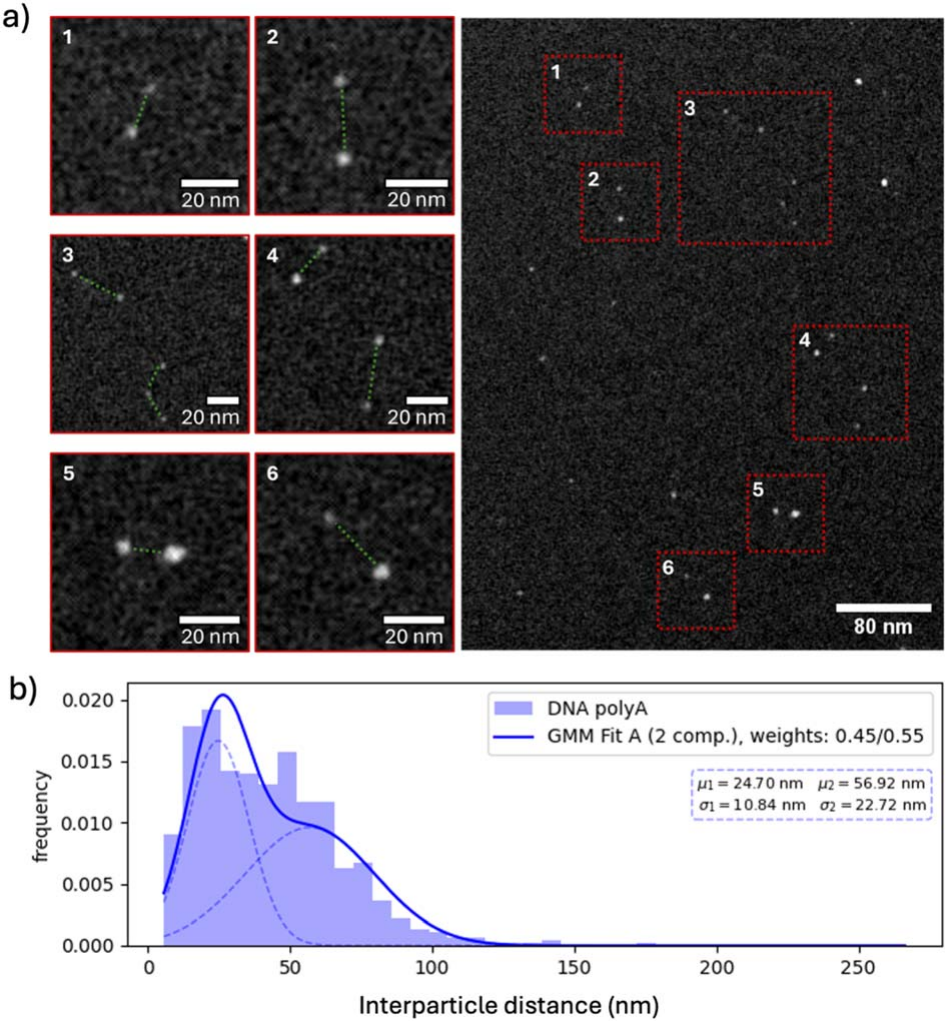
(a) HAADF-STEM images of AuNP@polyA in a liquid environment, showing the presence of dimers and individual particles. (b) Two-component Gaussian mixture model (GMM) showing the presence of AuNP@polyA duplexes and single AuNP@polyA.

Further characterization of AuNP@polyA was carried out using HAADF-STEM in colloidal dispersion, by using a commercial liquid cell (K-kit).^27,28^ This liquid cell is a silicon-based micro channel device which enables the characterization of nanoparticles in a liquid environment. HAADF-STEM measurements using a liquid cell showed the presence of numerous AuNP@polyA dimers in the liquid (Fig. 2), suggesting the formation of AuNP dimers through interparticle polyA duplexes. To support this hypothesis, we measured the distribution of nearest neighbour distances in the sample, expecting to find two distinct particle populations, namely particle pairs in close proximity and randomly distributed single particles. We thus performed a quantitative analysis using a two-component Gaussian mixture model (GMM). For the AuNP@PolyA distribution, the resulting component weights were approximately 0.453 and 0.547, indicating the presence of two comparably represented subpopulations, which is consistent with the interpretation of both duplex structures and randomly distributed particles being present (Fig. 2b and S6a). Furthermore, these images show distinct dimer lengths due to the presence of different polyA interaction sites.

**Fig. 3:**
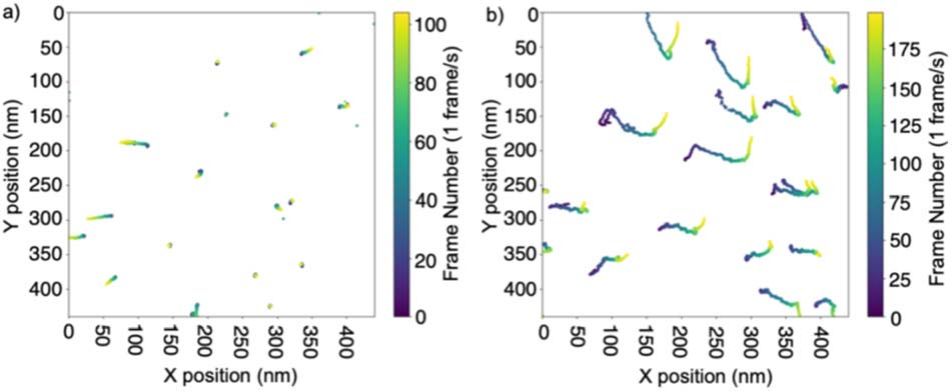
Scatter plots of the trajectories of the center position of AuNP@polyA. The center position of each particle is tracked across a time series of HAADF-STEM images with an electron dose of 20 e^−^ per Å^2^ per frame (a) and 50 e^−^ per Å^2^ per frame (b). When the electron dose is low, AuNP@polyA dimers move as stable units (a), whereas for the higher electron dose the polyA structure might be disrupted by the high energy electron beam, ultimately leading to fusion of AuNPs (b).

Interestingly, liquid-HAADF-STEM additionally offers the possibility of recording time series. When applying an electron dose of 20 e^−^ per Å^2^ per frame, dimers were found to be moving as stable units (see Fig. 3a, SI Videos S1 and S2). However, when the electron dose was increased above 50 e^−^ per Å^2^ per frame, a clear reduction in interparticle distance was observed, suggesting that the polyA structure might be disrupted by the high energy electron beam, ultimately leading to fusion of AuNPs (Fig. 3b).

**Fig. 4:**
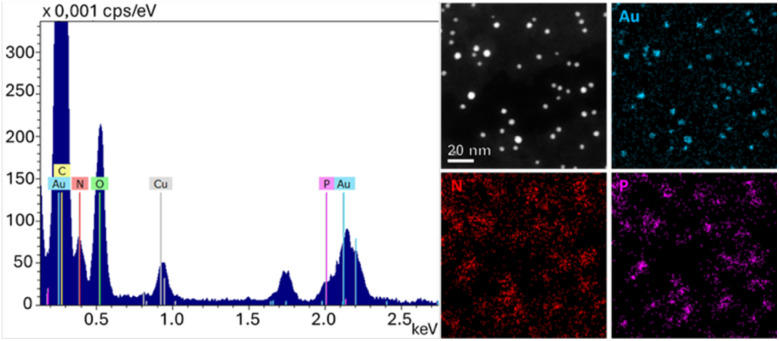
EDX spectra and elemental maps for AuNP@polyA under dry conditions, confirming the presence of N and P (from polyA) around AuNPs.

Additional characterization of AuNP@polyA conjugates was carried out using EDX measurements under dry conditions. The EDX spectrum and the elemental maps revealed the presence of nitrogen and phosphorus signals around AuNPs. These elements are part of the chemical structure of DNA and thus, their detection confirms the conjugation and localization of polyA around the AuNPs (Fig. 4).

### Preparation and characterization of AuNP@polyT

Experimental studies of different nucleotides indicate that adenine (A) has the highest affinity for the gold surface, whereas thymine (T) has the lowest affinity.^29^ Therefore, we carried out a control experiment analyzing the behavior of a different single stranded DNA, poly-thymine (polyT). The conjugation of thiolated-polyT (45 bases) to AuNPs (3.5 nm diameter) was carried out using the freezing protocol described above. As a result, AuNP@polyT (di-H_2_O, 1 μM of polyT on the Au surface) with an average of 13 polyT strands per particle was obtained (Fig. S2).

**Fig. 5:**
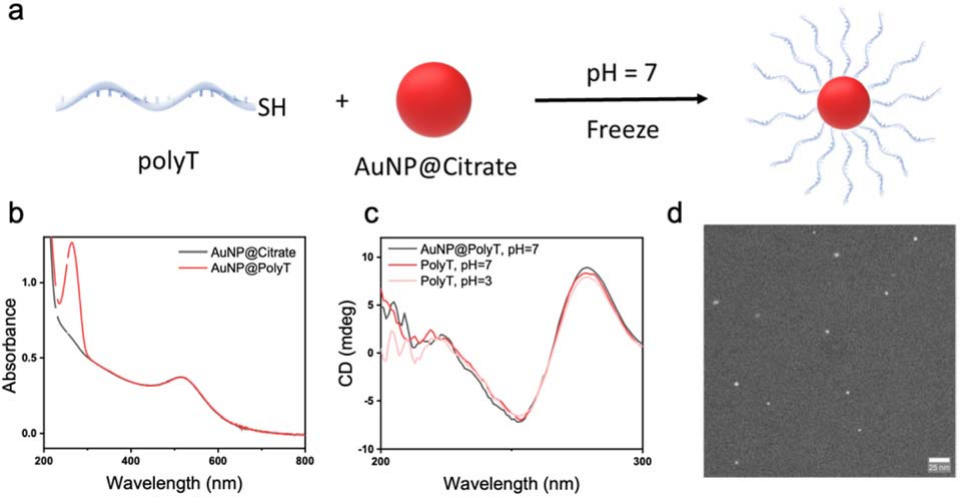
(a) Schematic illustration of AuNP@polyT conjugates. (b) UV-vis spectra of AuNP@citrate and AuNP@polyT. (c) CD spectra of AuNP@polyT at pH = 7 (black), polyT (red) at pH = 7 and polyT (pink) at pH = 3. (d) Representative liquid-phase TEM image of AuNP@polyT.

UV-vis spectra of AuNP@citrate and AuNP@polyT (1 μM of polyT on the Au surface) showed identical LSPR bands, confirming colloidal stability and the absence of AuNP aggregates (Fig. 5b). A typical DNA band in the UV region is observed upon polyT adsorption, on top of the standard spectrum of AuNPs. However, in contrast to the case of polyA conjugation, the CD spectra for AuNP@polyT (di-H_2_O, 1 μM of polyT, pH = 7) and sspolyT (di-H_2_O, 1 μM, pH = 7) show almost coincident patterns and intensities, suggesting the presence of isolated polyT strands on AuNPs (Fig. 5c). This result suggests that the conjugation of AuNP@polyT does not alter the secondary structure of polyT, thus retaining the same conformation as that of free polyT in solution.

Again, AuNP@polyT was characterized by liquid phase TEM. The images (Fig. 5d) revealed the presence of isolated AuNP@polyT (no dimers), with interparticle distances of 50 nm on average, thereby confirming the absence of PolyT hybridization between AuNPs. Further information can be obtained from liquid-phase TEM videos of AuNP@polyT samples diffusing in the aqueous solvent (SI Video S3). Statistical analysis of AuNP@polyT using a two-component Gaussian mixture model (GMM) reveals that a second distribution yielded component weights of approximately 0.200 and 0.800, suggesting that the distribution is dominated by a single population, with the minor component mostly including the tail of the distribution. The main component of the AuNP@polyT measurements also overlaps with the component of the AuNP@polyA measurements, which is consistent with the randomly distributed particles. These results support the conclusion that the distribution represents predominantly randomly positioned AuNPs (Fig. S6b). We additionally recorded scatter plots of the trajectories of the center position of AuNP@polyT. The center position of each particle was tracked across a time series of HAADF-STEM images with an electron dose of 20 e^−^ per Å^2^ per frame (Fig. S7). We observe that AuNP@polyA (dimers) diffuse slower than AuNP@polyT (single particles), as expected.

### Effect of external stimuli on AuNP@polyA

**Fig. 6:**
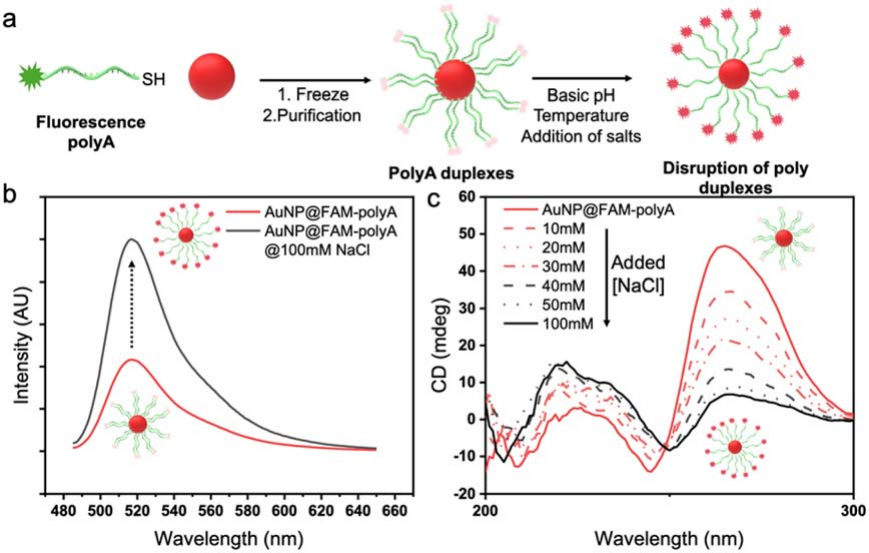
(a) Schematic illustration of the behavior of AuNP@polyA duplexes in the presence of added NaCl. (b) Fluorescence spectra of AuNP@FAM-polyA before and after addition of NaCl (100 mM). (c) CD spectra of AuNP@polyA at different NaCl concentrations, as labeled.

A-motif duplexes are stabilized by protonated AH^+^–H^+^A units *via* both reverse Hoogsteen bonding and electrostatic interactions between positively charged adenines and negatively charged phosphate groups of the backbone,^21^ which however can be disrupted by external stimuli (*e.g.* pH, NaCl, temperature…, see Fig. S8). It is well known that the presence of duplexes in fluorescent FAM-polyA promotes self-quenching of the fluorescence intensity,^30^ offering the possibility to monitor the disruption of polyA duplexes *via* recovery of the fluorescence signal (Fig. 6a).

The conjugation of thiolated-FAM-polyA (45 bases, 2 μM) with AuNPs (3.5 nm, 100 nM) was carried out following the protocol described above. Stimuli-responsive studies of AuNP@FAM-polyA were carried out by addition of different amounts of NaCl (5 M) as an external stimulus. Subsequent fluorescence measurements of AuNP@FAM-polyA (di-H_2_O, 1 μM of DNA on the Au surface) showed a strong enhancement of the intensity upon addition of NaCl, indicating the disruption of polyA duplexes (Fig. 6a and b). Moreover, CD measurements on AuNP@polyA after NaCl addition revealed changes in the polyA conformation, from duplexes into individual polyA strands (Fig. 6c). Similar experiments were carried out with AuNP@polyT, in which the addition of different amounts of NaCl (5 M) did not lead to significant changes in the CD spectra for polyT, indicating that their secondary structure was not altered by the presence of NaCl (Fig. S9).

**Fig. 7:**
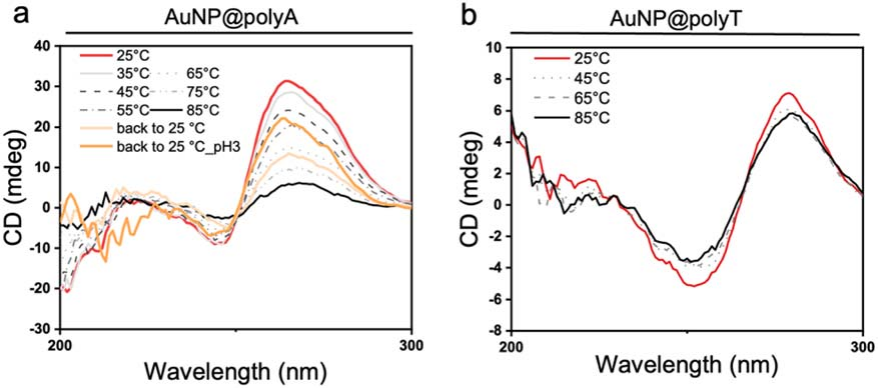
CD spectra at different temperatures for AuNP@polyA (a) and AuNP@polyT (b).

We additionally studied the effect of temperature on AuNP@polyA (di-H_2_O, 1 μM of DNA on the Au surface), expecting that a higher temperature would lead to the disruption of non-covalent interactions between polyA strands in polyA duplexes. A strong decrease of CD intensity was indeed registered when temperature was increased, confirming a conformational change in polyA. While room temperature CD spectra revealed the presence of polyA duplexes, at temperatures above 65 °C the duplexes were disrupted and polyA single strands were present on the AuNP surface. This conclusion was validated by the reversibility of the process, as evidenced by partial recovery of the CD signal when polyA duplexes were formed at room temperature and when the pH was adjusted to 3 (Fig. 7a and Fig. S10). On the other hand, variable-temperature CD experiments were carried out with AuNP@polyT (di-H_2_O, 1 μM of DNA on the Au surface). In this case, CD spectra recorded at different temperatures showed similar traces, suggesting the presence of the same polyT conformation (single strands) when anchored on AuNPs (Fig. 7b).

### Effect of grafting density on the formation of AuNP@polyA duplexes

**Fig. 8:**
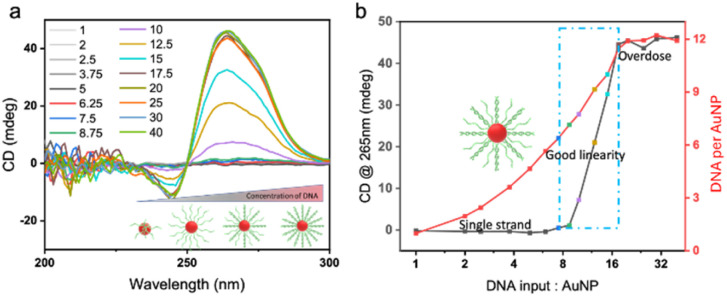
(a) CD spectra of AuNP@polyA prepared at different polyA/AuNP ratios. (b) Correlation between the number of polyA strands on AuNPs and CD intensity at 265 nm.

Next, we studied the relationship between the concentration of ssDNA on AuNPs and the formation of stable polyA duplexes. For this purpose, we carried out the conjugation using different ratios between polyA and AuNPs, from 1 to 40. Interestingly, chiroptical studies of AuNP@polyA revealed null CD spectra at low polyA/AuNP ratios (from 1 to 7.5), with a maximum of 6 polyA strands per AuNP (Fig. 8a). In these cases, polyA strands might wrap around the AuNP due to their high affinity to the gold surface, thereby avoiding the formation of duplexes.^31,32^ On the other hand, at higher polyA/AuNP ratios (from 7.5 to 16) a linear increase of the CD signal was observed due to the formation of polyA duplexes on AuNPs (Fig. 8b). Additionally, the variation in fluorescence intensity matched well with that of CD intensity, further suggesting the strong relationship between polyA conformation and CD intensity (Fig. S11). Interestingly, a maximum of CD intensity was obtained when the polyA/AuNP ratio was 16 (12 polyA strands per AuNP based on quantification), suggesting the formation of six polyA duplexes on each AuNP.

Similar experiments were carried out using different polyT/AuNP ratios, observing a linear increase of CD intensity from lower to higher ratios but the same CD trace, which is indicative of the absence of thymine secondary structure on gold surfaces (Fig. S12). From these experiments, we can conclude that the formation of polyA duplexes on AuNPs is strongly dependent on the concentration of polyA on the AuNP surface; higher concentrations of polyA promote the formation of duplexes, whereas low concentrations of polyA promote the interaction of polyA strands with the gold surface preventing the formation of polyA duplexes.

## Conclusions

By using a combination of spectroscopic techniques (CD, UV-vis, fluorescence emission) and TEM in liquid, we determined the specific interactions and conformations of polyA and polyT when adsorbed onto small AuNPs. From these studies, we confirmed the presence of polyA duplexes but isolated polyT strands conjugated with AuNPs. Upon use of external stimuli (NaCl and temperature) the secondary structure of AuNP@polyA could be manipulated, from duplexes to isolated polyA chains. Interestingly, liquid STEM images and videos confirmed the presence of AuNP@polyA dimers compatible with nanoparticles connected by polyA duplexes. Finally, we studied the effect of polyA density and we found a strong dependency on DNA density, with higher numbers of polyA chains on AuNPs promoting the formation of duplexes.

By the combined use of spectroscopic and advanced electron microscopy techniques, relevant information can be obtained about DNA interactions and conformations on AuNPs. These studies on the DNA structure and its interactions with NPs should be considered when designing sensors and contrast agents based on DNA-AuNPs.

## Author contributions

J. D., I. G. and M. N.-M. performed experimental synthesis, characterization, and data analysis. B. D. B., N. C. and S. B. performed liquid STEM experiments and analysis. J. D., I. G., and M. N.-M. wrote the original draft. L. L. M. supervised the whole project, writing, review, and editing. All the authors received and approved the final manuscript.

## Conflicts of interest

There are no conflicts to declare.

## Supplementary Material

SC-016-D5SC04459F-s001

SC-016-D5SC04459F-s002

SC-016-D5SC04459F-s003

SC-016-D5SC04459F-s004

## Data Availability

The data underlying this study are available in the published article and its SI: Table S1, Scheme S1 and Fig. S1–S13. See DOI: https://doi.org/10.1039/d5sc04459f. If required, after acceptance of the paper, raw data will be made publicly available at an open repository such as Zenodo.
