# Supplementary material for: Distinct structural interactions of polyadenine and polythymine on gold nanoparticles: from single strands to duplexes
Source: Chem Sci. 2025 Jul 31;16(35):15947–54. doi: 10.1039/d5sc04459f (PMC12333531; doi:10.1039/d5sc04459f)
Supplement: SC-016-D5SC04459F-s001 [file SC-016-D5SC04459F-s001.pdf]

Supplementary Information (SI) for Chemical Science.
This journal is © The Royal Society of Chemistry 2025

Supplementary Information

Distinct Structural Interactions of polyAdenine and polyThymine on Gold Nanoparticles: From Single Strands to Duplexes

Manuel Núñez-Martínez[a,†,*], Jinyi Dong[a,†], Isabel García[a,b,*], Bjorn De Busschere[d], Nathalie Claes[d], Sara Bals[d,*], Luis M. Liz-Marzán[a,b,c,*]

[a]CIC biomaGUNE, Basque Research and Technology Alliance (BRTA), 20014, Donostia-San Sebastián (Spain).

[b]Networking Biomedical Research Center, Bioengineering, Biomaterials and Nanomedicine (CIBER-BBN), 20014, Donostia-San Sebastián (Spain)

[c]Ikerbasque, 48009, Bilbao (Spain)

[d]EMAT and NANOlab Center of Excellence, University of Antwerp, B-2020 Antwerp, Belgium

[e] CAS Key Laboratory of Nano-Bio Interface, Suzhou Key Laboratory of Functional Molecular Imaging Technology, Division of Nanobiomedicine and i-Lab, Suzhou Institute of Nano-Tech and Nano-Bionics, Chinese Academy of Sciences, Suzhou, 215123 China

[†]These authors contributed equally.

*Corresponding authors' email: mnunez@cicbiomagune.es (M.N.-M.)
igarcia@cicbiomagune.es (I.G.)
sara.bals@uantwerpen.be (S.B.)
llizmarzan@cicbiomagune.es (L.M.L.-M.)

Table S1. Sequences of DNA oligonucleotides employed in this work.

Oligos	Sequences and modifications (from 5` to 3`)
polyA	[Thiol C6]-AAAAAAAAAAAAAAAAAAAAAAAAAAAAAAAAAAAAAAAAAAAAA
FAM-polyA	[Thiol C6]-AAAAAAAAAAAAAAAAAAAAAAAAAAAAAAAAAAAAAAAAAAAAA-[FAM]
Non thiolated_A45	AAAAAAAAAAAAAAAAAAAAAAAAAAAAAAAAAAAAAAAAAAAAA
polyT	[Thiol C6]-TTTTTTTTTTTTTTTTTTTTTTTTTTTTTTTTTTTTTTTTTTTTT
FAM-polyT	[Thiol C6]-TTTTTTTTTTTTTTTTTTTTTTTTTTTTTTTTTTTTTTTTTTTTT-[FAM]
A11	[Thiol C6]-AAAAAAAAAAA
A22	[Thiol C6]-AAAAAAAAAAAAAAAAAAAAAA

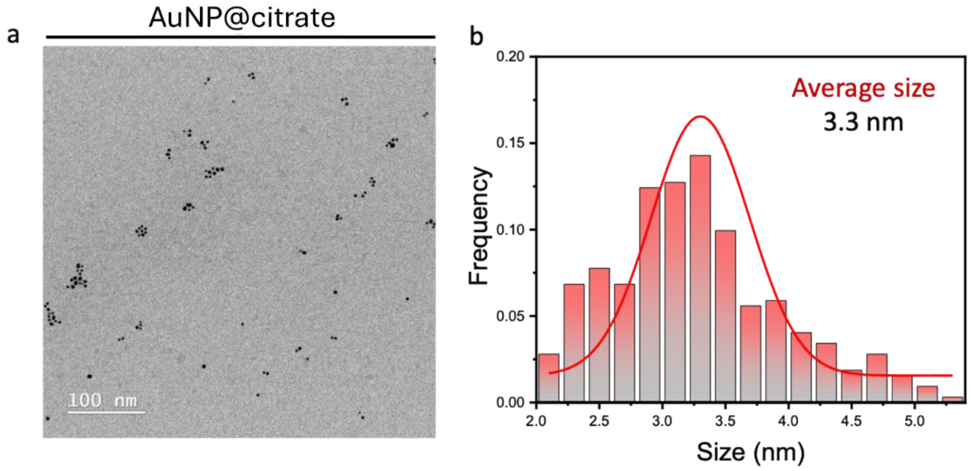

Figure S1. a) TEM image of dried AuNP@citrate and corresponding size distribution histogram (b).

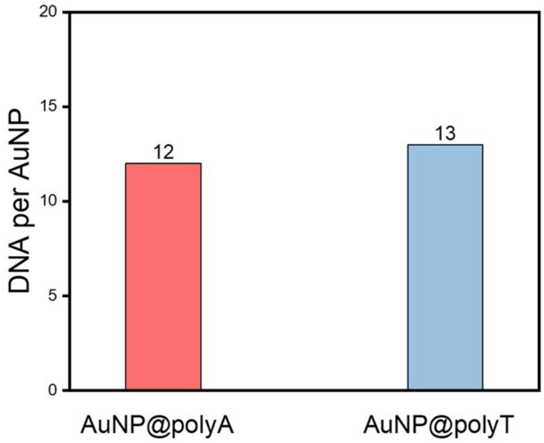

Figure S2. Fluorometric quantification of thiolated polyA and thiolated polyT on 3.5 nm AuNP.

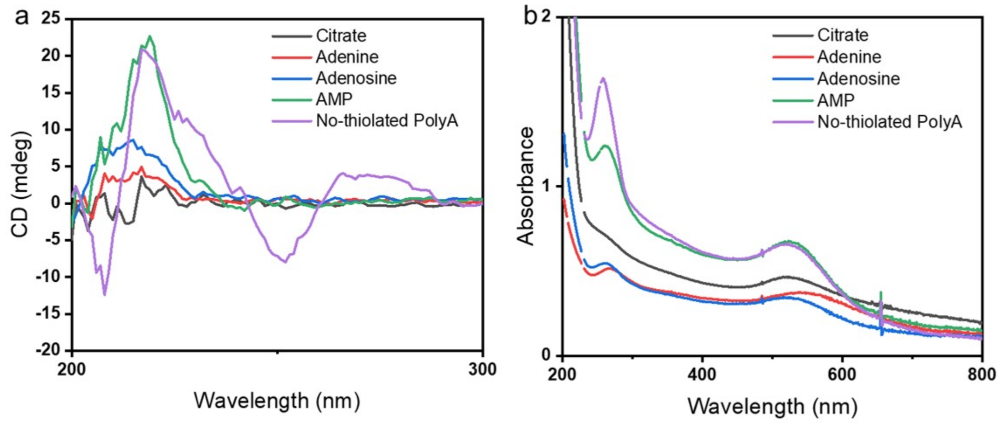

Figure S3. Freeze-driven adsorption of different adenine-containing molecules on 100 nM AuNPs (3.5 nm). The concentration of citrate was 2.2 mM, those of adenine, adenosine and adenosine monophosphate (AMP) were 90 uM. Non-thiolated A45 was 2 μM, containing 90 μM adenine. Circular dichroism (a) and UV-Vis absorbance (b) spectra of AuNPs with adenine-containing molecules after purification.

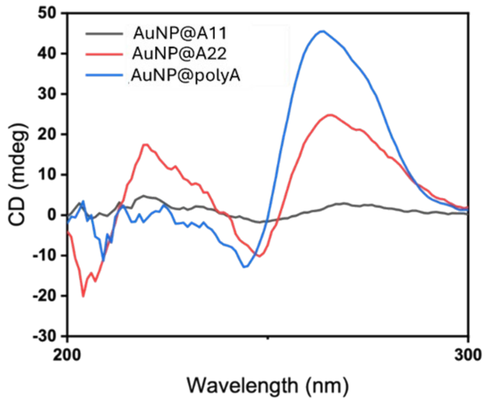

Figure S4. CD spectra of AuNPs functionalized with sspolyA containing different number of adenines: AuNP@A11 (black), AuNP@A22 (red), AuNP@polyA (blue).

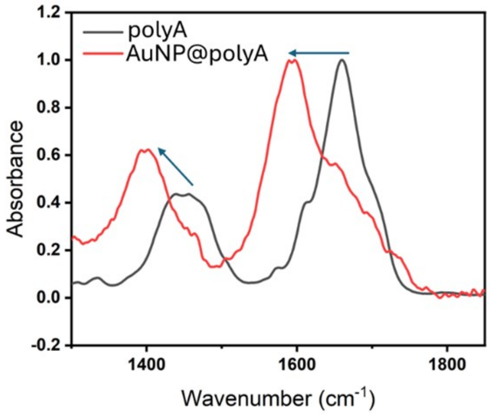

Figure S5. FT-IR spectra of polyA (black) and AuNP@polyA (red).

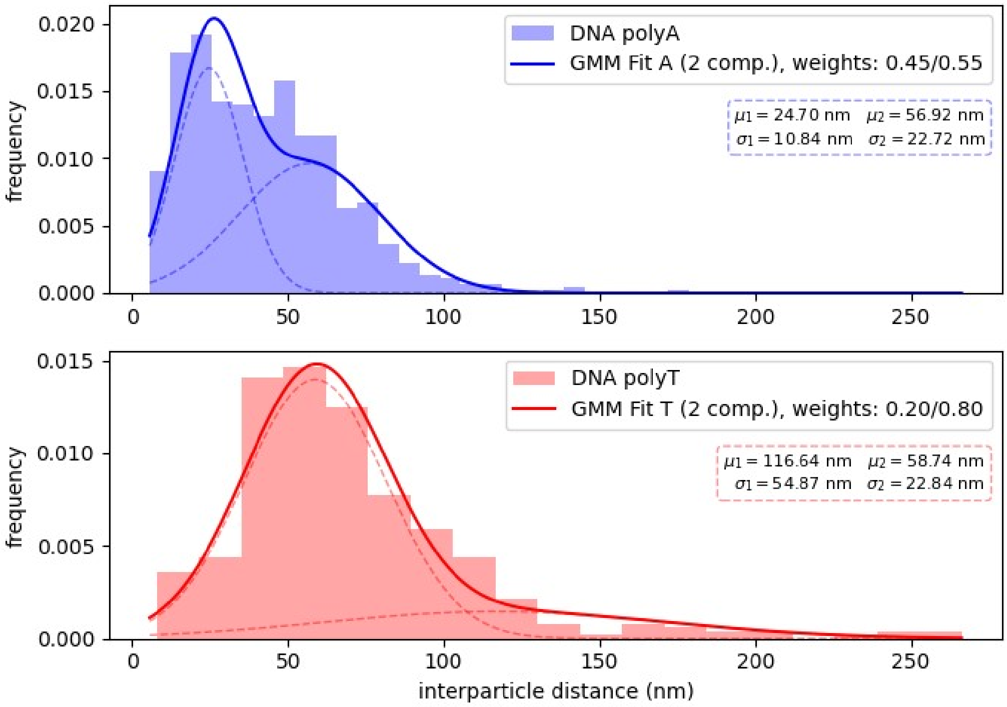

Figure S6. To support the hypothesis that the observed nearest neighbour distance distribution of the PolyA sample arose from two distinct particle populations, namely, particle pairs in close proximity and randomly distributed single particles, while the PolyT distribution consisted solely of measurements of randomly distributed particles, we performed a quantitative analysis using a two-component Gaussian mixture model (GMM). Both distributions were fitted with a model comprising two Gaussian components. For the PolyA distribution, the resulting component weights were approximately 0.453 and 0.547, indicating the presence of two comparably represented subpopulations, consistent with the interpretation of both duplex structures and randomly distributed particles being present. In contrast, the second distribution yielded component weights of approximately 0.200 and 0.800, suggesting that the distribution is dominated by a single population, with the minor component mostly including the tail of the distribution. The main component of the PolyT measurements also overlaps with the component of the PolyA measurements that is consistent with the randomly distributed particles. These results support the conclusion that the second distribution represents predominantly randomly positioned particles, while the first captures a mixture of particle configurations.

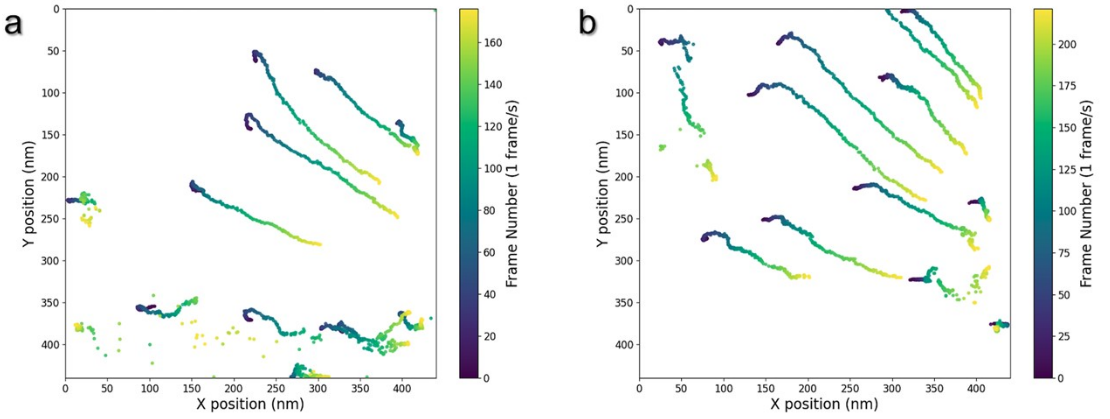

Figure S7. Scatter plots of the resulting trajectories of the centre position of AuNP@polyT. The centre position of each particle is tracked across a time series of HAADF-STEM images with an electron dose of 20 $e^-/Å^2$ per frame. For AuNP@polyT the particles show a higher mobility in comparison to AuNP@polyA.

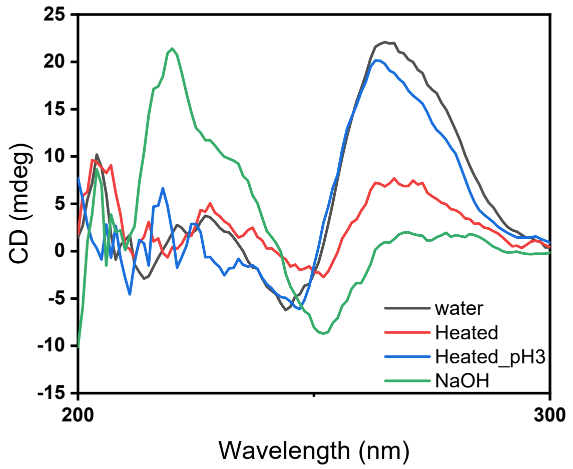

Figure S8. CD spectra of AuNPs@polyA in water solution (black line), after heating (red line), after heating and pH =3 (blue line) and in basic solution (green).

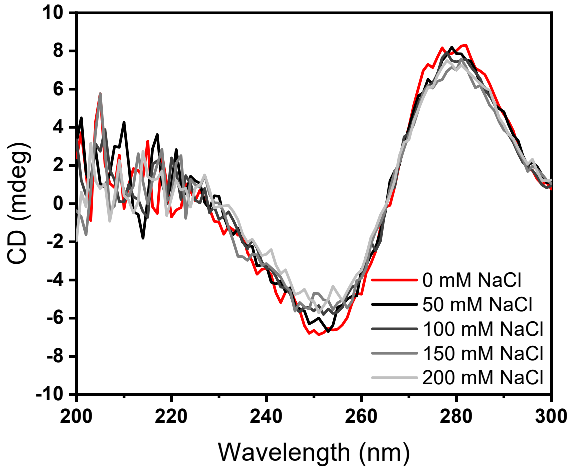

Figure S9. CD spectra of AuNP@polyT after addition at different concentrations of NaCl as labelled.

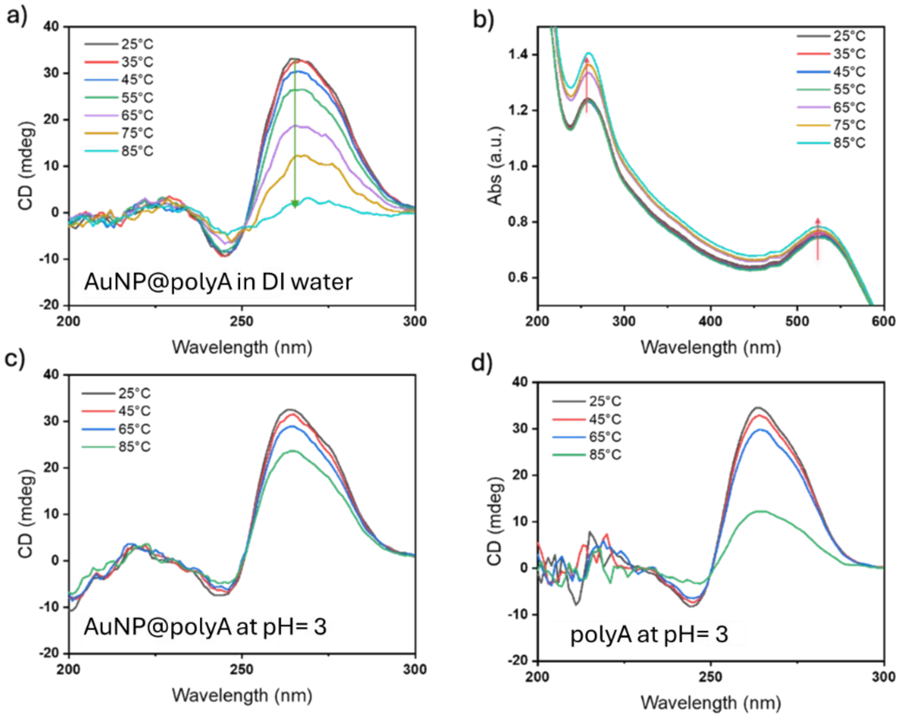

Figure S10. a,b) Circular dichroism (a) and UV-vis (b) spectra of AuNPs@polyA at different temperatures, as labeled. c,d) Circular dichroism spectra of AuNP@polyA (c) and polyA at pH=3 (d).

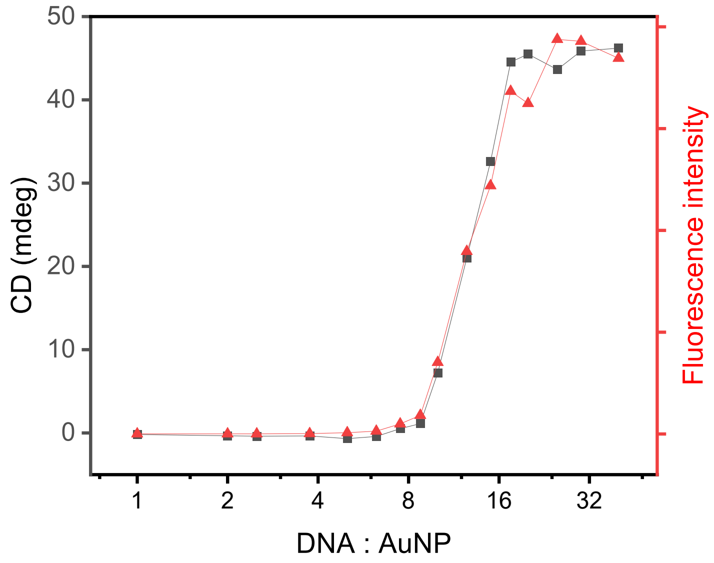

Figure S11. Correlation between the fluorescence of AuNP@polyA and CD intensity at 265 nm.

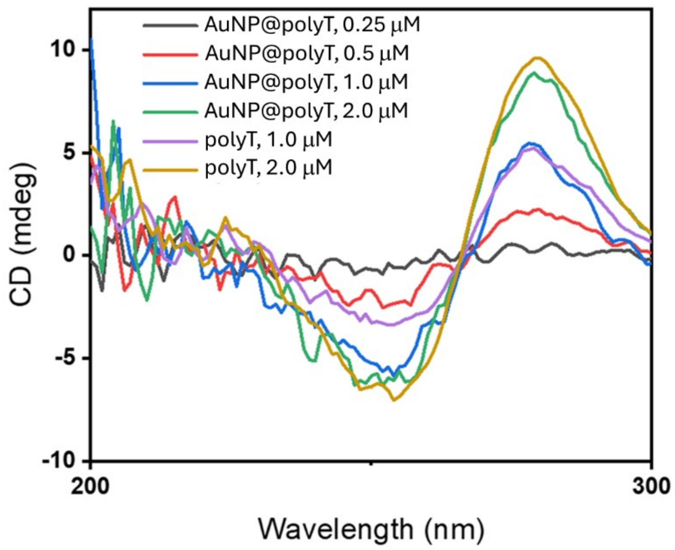

Figure S12. Circular dichroism spectra of AuNP@polyT prepared at different DNA/AuNP ratios, as labelled.

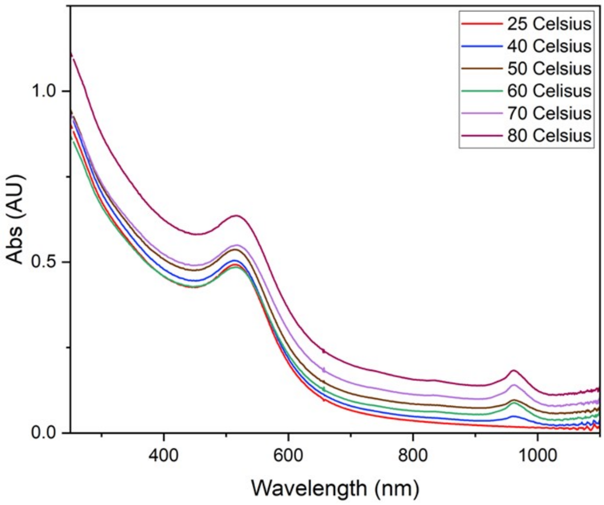

Figure S13. UV-Vis studies of AuNP@citrate at different temperatures.

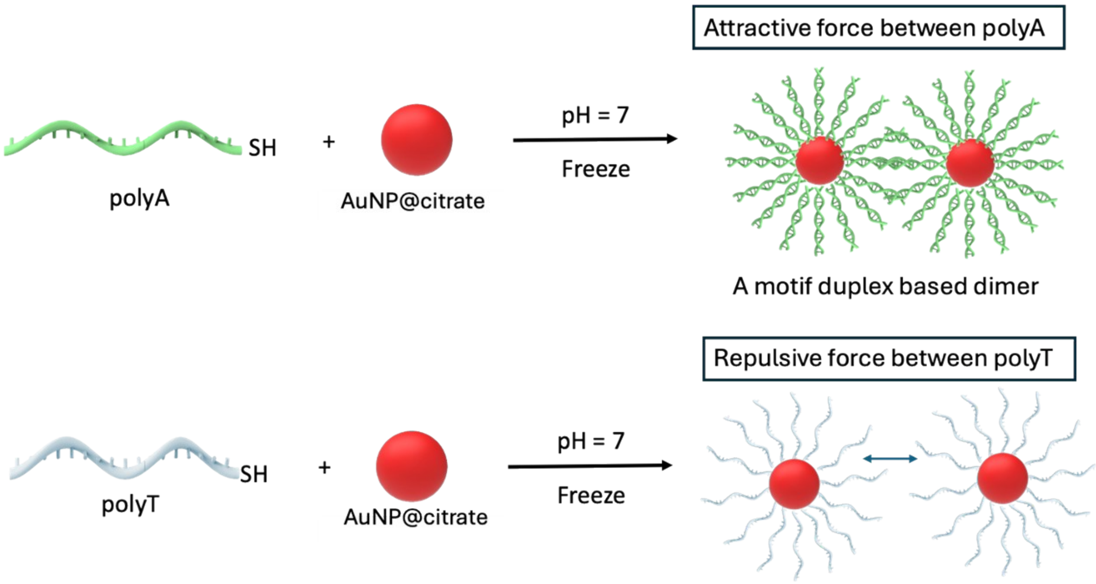

Scheme S1. Schematic illustration of the formation mechanism of AuNP@polyA dimers and AuNP@polyT with single polyT strands.
